# Comparative evaluation of composite inflammatory indices for predicting prostate cancer risk and mortality: a NHANES-based study with external validation

**DOI:** 10.3389/fonc.2026.1830463

**Published:** 2026-05-11

**Authors:** Hengxin Chen, Jiahao Lei, Wenzhuo Zhang, Zongren Wang, Ren Liu, Lingwu Chen

**Affiliations:** Department of Urology, The First Affiliated Hospital of Sun Yat-sen University, Guangzhou, China

**Keywords:** composite inflammatory indices, NHANES, prognosis, prostate cancer, risk stratification

## Abstract

**Background:**

Prostate cancer (PCa) is one of the most common malignancies in men, and chronic inflammation has been implicated in its initiation and progression. Composite inflammatory indices, based on routine peripheral blood parameters, reflect systemic inflammatory and immune status. However, their clinical utility for risk stratification and prognostic evaluation in PCa remains unclear.

**Methods:**

Data from the National Health and Nutrition Examination Survey (NHANES) 1999–2018 were analyzed, including 14,562 participants. Associations between 14 composite inflammatory indices and both PCa risk and all-cause mortality risk were evaluated using multivariable logistic regression and Cox proportional hazards models. Generalized estimating equations, generalized additive models, and restricted cubic splines were used to explore robustness and nonlinear associations. Kaplan-Meier survival analysis was used to evaluate prognostic associations. Furthermore, a Chinese hospital-based cohort was used for external validation of key inflammatory indices. Machine learning models were used to assess prognostic predictive performance.

**Results:**

Several composite inflammatory indices were significantly associated with PCa risk. Increased systemic immune-inflammation index (SII), platelet-to-lymphocyte ratio (PLR), and neutrophil-to-lymphocyte ratio (NLR) were related to higher PCa risk, while elevated lymphocyte-to-monocyte ratio (LMR), prognostic nutritional index (PNI), hemoglobin–albumin–lymphocyte–platelet (HALP), and monocyte-to-high-density lipoprotein cholesterol ratio (MHR) were associated with reduced risk. Restricted cubic spline analysis indicated that some inflammatory indices exhibited potential non-linear relationships with PCa incidence and all-cause mortality risk. Regarding prognosis, increased neutrophil-to-platelet ratio (NPR), systemic inflammation response index (SIRI), and aggregate index of systemic inflammation (AISI) were associated with a higher risk of all-cause mortality, while high C-reactive protein-to-albumin ratio (CAR), creatinine-to-lymphocyte ratio (CLR), and inflammatory burden index (IBI) were linked to poorer prognosis. The external cohort further validated the stable association of SII, PLR, and NLR with adverse prognosis. Machine learning analyses yielded consistent discriminatory performance, supporting the prognostic relevance of inflammation-related hematological features.

**Conclusion:**

Multiple composite inflammatory indices are associated with the PCa risk and prognosis. Among them, SII, PLR, and NLR demonstrated stable predictive value for adverse prognosis across different populations. Inflammatory indices based on peripheral blood parameters can contribute to risk stratification and clinical decision-making for PCa patients.

## 1 Introduction

Prostate cancer (PCa) is the second most common malignancy among men worldwide and a significant cause of cancer-related deaths (1). Despite advances in early detection and comprehensive treatment in recent years, the burden of PCa and its long-term survival still faces many challenges. The occurrence and progression of PCa are recognized as multifactorial processes involving genetic susceptibility, metabolic disorders, lifestyle, and chronic inflammation (2, 3). The role of inflammatory responses in the formation of the tumor microenvironment and tumor progression is gradually gaining attention. Accumulating evidence indicates that systemic inflammatory status may serve as a valuable indicator for cancer risk stratification and prognostic assessment, providing novel insights into the diagnosis and treatment of PCa (4–6).

Inflammation is an essentially protective immune response of the body to maintain tissue homeostasis and defend against pathogen invasion (7, 8). However, persistent or dysregulated inflammation can induce a pro-tumorigenic microenvironment through mechanisms like oxidative stress and tissue remodeling, thereby creating favorable conditions for tumorigenesis and progression (9). In recent years, inflammatory indices based on routine peripheral blood counts and biochemical parameters have been widely used to reflect the body’s systemic inflammatory and immune status. These indices can be divided into single-parameter and composite inflammatory indices, the latter integrating multiple parameters. Representative composite indices include the aggregate index of systemic inflammation (AISI), C-reactive protein–to-albumin ratio (CAR), platelet-to-lymphocyte ratio (PLR), C-reactive protein–to-lymphocyte ratio (CLR), hemoglobin–albumin–lymphocyte–platelet (HALP) score, prognostic nutritional index (PNI), inflammatory burden index (IBI), lymphocyte-to-monocyte ratio (LMR), systemic immune–inflammation index (SII), monocyte-to–high-density lipoprotein cholesterol ratio (MHR), neutrophil-to-lymphocyte ratio (NLR), systemic inflammation response index (SIRI), neutrophil-to-platelet ratio (NPR), and platelet-to-albumin ratio (PAR). Many of these have been confirmed as effective markers for assessing systemic inflammation (10–14). Owing to their accessibility, non-invasive nature, and low cost, these indices are increasingly recognized as practical biomarkers in oncological research. Growing evidence indicates that composite inflammatory indices are associated not only with cancer incidence but also with tumor progression, survival outcomes, and treatment responses (15–18).

The National Health and Nutrition Examination Survey (NHANES), conducted by the Centers for Disease Control and Prevention (CDC), is a continuous, population-based cross-sectional survey. The NHANES collects nationally representative data from the U.S. population through a complex, multi-stage, stratified sampling design. The data encompasses demographic characteristics, lifestyle, medical history, physical examination, and laboratory measurements, including complete blood counts and biochemical parameters. Analyzing NHANES data can elucidate the relationship between inflammatory indices and PCa risk and mortality.

Although existing studies have partially revealed the important role of inflammation in tumorigenesis and progression, research on the relationship between multiple composite inflammatory indices and PCa remains scarce. Furthermore, previous studies focused on single inflammatory index, overlooking the potential synergy of different inflammatory indices, leading to an underestimation of their overall predictive value. In addition, traditional approaches may be limited in capturing complex, non-linear relationships among correlated inflammatory and clinical variables. Therefore, complementary analytical strategies that integrate multiple hematological and clinical features may provide additional insights into individualized risk stratification. This study aimed to comprehensively evaluate the associations between multiple composite inflammatory indices (including AISI, CAR, PLR, CLR, HALP, PNI, IBI, LMR, SII, MHR, NLR, SIRI, NPR, and PAR) and PCa risk and all-cause mortality. Subsequently, an independent cohort from a Chinese hospital is used for external validation of the primary findings. By integrating population-based data with real-world clinical evidence and utilizing multilevel statistical models to rigorously control for potential confounding factors, this study seeks to clarify the clinical value of inflammatory indices in PCa risk stratification and prognostic evaluation.

## 2 Methods

### 2.1 Study design and data source

This study is a population-based observational study integrating cross-sectional data with prospective follow-up information. The primary analysis data were derived from the NHANES. We integrated data from five 2-year NHANES cycles spanning 1999 to 2018. The National Center for Health Statistics Research Ethics Review Board approved NHANES, and all participants provided written informed consent. Furthermore, to validate the robustness and generalizability of the main findings, an independent external validation cohort from the First Affiliated Hospital of Sun Yat-sen University was included to assess the prognostic predictive value of key inflammatory indices in a real-world clinical setting. All procedures were approved by the Ethics Committee of the First Affiliated Hospital of Sun Yat-sen University [Approval No (2025): 920].

### 2.2 Inclusion and exclusion criteria

#### 2.2.1 NHANES study population

We initially included all male participants with complete questionnaires, physical examination data, and laboratory test information. Inclusion criteria included: age ≥ 20 years; availability of complete peripheral blood routine and biochemical indicators required for calculating the inflammatory indices; availability of definitive history of cancer; availability of complete follow-up and outcome information.

Exclusion criteria included: all female participants; lacking key laboratory indicators; lacking cancer diagnosis or follow-up information; suffering from tumors other than PCa. After strict screening, we ultimately included 14,562 participants from a total of 102,956 individuals, among whom there were 423 PCa patients and 14,139 non-PCa control group.

#### 2.2.2 External validation cohort

The external validation cohort was collected from the First Affiliated Hospital of Sun Yat-sen University, including 200 PCa patients diagnosed between 2016 and 2020. All patients were pathologically confirmed as PCa after undergoing prostate needle biopsy and subsequently underwent radical prostatectomy. All patients underwent routine blood collection and were recorded within 3 days before the biopsy, and complete follow-up and survival information were available. During the follow-up, a total of 61 death events were observed. This cohort was used to validate the association between inflammatory indices and PCa prognosis.

### 2.3 Definition of prostate cancer and outcome assessment

For the NHANES data, cancer history was obtained through a standardized questionnaire. Participants were asked: “Have you ever been told by a doctor or other health professional that you had cancer or a malignancy of any kind?” If they answered “Yes,” they were further asked, “What kind of cancer was it?” Then the cancer type was recorded. Outcome information was sourced from the follow-up database provided by the NCHS, and the cutoff for follow-up time was December 31, 2019. The primary outcomes included survival status and time (months) since the start of follow-up. In the external validation cohort, follow-up was conducted by telephone, and the cutoff for follow-up time was December 31, 2025.

### 2.4 Definition and calculation of inflammation indices

Composite inflammatory indices were calculated based on routine peripheral blood counts and biochemical parameters, including neutrophil count (×10^9^/L), lymphocyte count (×10^9^/L), monocyte count (×10^9^/L), platelet count (×10^9^/L), hemoglobin (g/L), serum albumin (g/L), high-density lipoprotein cholesterol (mg/dL), and C-reactive protein (mg/L). The calculation formulas are as follows:

- AISI = (Platelet count × Neutrophil count × Monocyte count) / Lymphocyte count
- CAR = C-reactive protein level / Albumin level
- PLR = Platelet count / Lymphocyte count
- CLR = C-reactive protein level / Lymphocyte count
- HALP = (Hemoglobin level × Albumin level × Lymphocyte count) / Platelet count
- PNI = Albumin level + (5 × Lymphocyte count)
- IBI = (C-reactive protein level × Neutrophil count) / Lymphocyte count
- LMR = Lymphocyte count / Monocyte count
- SII = (Platelet count × Neutrophil count) / Lymphocyte count
- MHR = Monocyte count / High-density lipoprotein cholesterol level
- NLR = Neutrophil count / Lymphocyte count
- SIRI = (Neutrophil count × Monocyte count) / Lymphocyte count
- NPR = Neutrophil count / Platelet count
- PAR = Platelet count / Albumin level

### 2.5 Definition of covariates

Covariates included demographic characteristics and health-related factors. Demographic characteristics included age, race (Mexican American, Non-Hispanic White, Non-Hispanic Black, Other Hispanic, Other Race), and education level (less than high school, high school or equivalent, or some college or higher). Health-related factors included body mass index (BMI, categorized as Normal, Overweight, Obese), smoking history, alcohol use, and histories of hypertension and diabetes.

### 2.6 Statistical analysis

All analyses accounted for the complex sampling design using sampling weights of NHANES. Data processing and analysis were performed using R software (http://www.R-project.org, version 4.3.3). A two-sided P-value< 0.05 was considered statistically significant.

#### 2.6.1 Baseline characteristics analysis

Continuous variables were described as mean ± standard error or median (interquartile range). Differences between groups were assessed using the t-test for normally distributed data and the Mann-Whitney U test for non-normally distributed data. Categorical variables were presented as frequencies and percentages and compared using the chi-square test.

#### 2.6.2 Association between inflammatory indices and PCa risk

Inflammatory index was categorized into quartiles (Q1-Q4) based on its distribution in the population, with the lowest quartile (Q1) serving as the reference. Logistic regression models were used to assess the association between inflammatory indices and PCa risk, with three progressively adjusted models constructed: Model 1 adjusted for age; Model 2 adjusted for age, race, and education level; Model 3 further adjusted for BMI, smoking history, alcohol use, hypertension, and diabetes based on Model 2. Results were reported as odds ratios (OR) with 95% confidence intervals (CI), and trend test was performed to assess dose-response relationships. Considering the potential cluster effects, the generalized estimating equations (GEE) model was employed in sensitivity analysis. Generalized additive models (GAM) and smoothed curve fitting were used to explore potential non-linear associations between inflammatory indices and PCa risk. To evaluate the reliability of the current sample size, *post-hoc* power analysis was performed to calculate the statistical power. To avoid multicollinearity among highly correlated inflammatory indices, each index was entered into the regression models separately rather than simultaneously.

#### 2.6.3 Analysis of inflammatory indices and mortality risk

Among PCa patients, Cox proportional hazards regression models were used to evaluate the association between inflammatory indices and all-cause mortality risk. To explore potential non-linear associations, restricted cubic spline (RCS) modeling was performed based on the fully adjusted multivariable model. Knots were set at the 5th, 35th, 65th, and 95th percentiles of their distribution. The effective degrees of freedom (edf) were calculated by comparing goodness-of-fit between models with and without non-linear terms, and the likelihood ratio tests were used to assess the statistical significance of non-linear trends. For the NHANES data, the model construction was consistent with the risk analyses. For the validation cohort, the model construction was adjusted: Model 1 adjusted for age; Model 2 adjusted for age, BMI, smoking history, and alcohol use; Model 3 further adjusted for hypertension and diabetes based on Model 2. The proportional hazards assumption was evaluated using Schoenfeld residual tests. Given the exploratory nature of evaluating multiple correlated inflammatory indices, no formal multiplicity correction was applied. Instead, we implemented a structured analytic strategy emphasizing (i) consistency of effect estimates across models, (ii) magnitude and precision of associations (ORs/HRs with 95% CIs), and (iii) independent validation of key findings in an external cohort, which served as a confirmatory assessment to reduce the likelihood of spurious associations arising from multiple comparisons.

#### 2.6.4 Survival analysis and predictive performance assessment

Participants were grouped based on the median value of each inflammatory index, and Kaplan-Meier survival curves were plotted, with Log-rank tests employed to compare survival differences. In the validation cohort, time-dependent receiver operating characteristic (ROC) curves and calibration curves were further used to evaluate the predictive performance of the inflammatory indices.

#### 2.6.5 Machine learning-based predictive modeling

To further assess the predictive performance of different combinations of clinical characteristics and peripheral blood cell parameters, machine learning-based models were constructed as a complementary analysis. Four commonly used classification algorithms were implemented, including logistic regression, random forest (RF), support vector machine (SVM) with radial basis function kernel, and extreme gradient boosting (XGBoost) (19). Multiple feature sets were evaluated, including a clinical model (age, BMI, and smoking history) and a blood cell model (neutrophil, lymphocyte, and platelet counts), and combined models integrating clinical variables with blood cell parameters. For each algorithm and feature combination, predicted risk scores were generated and evaluated using time-dependent ROC analysis at 36 and 60 months. Model performance was quantified by the area under the ROC curve (AUC), and internal robustness was assessed using bootstrap resampling (100 iterations) to estimate mean AUC values and corresponding 95% confidence intervals. Comparative visualization of model performance across different algorithms and feature sets was performed at both time points.

To further compare algorithmic performance within a fixed feature set, ROC curves at 36 and 60 months were generated for logistic regression, RF, SVM, and XGBoost models based on the combined clinical and blood cell variables. In addition, XGBoost models trained on the NHANES dataset were externally validated using an independent clinical cohort, with predictive performance evaluated by time-dependent AUCs. To enhance model interpretability, SHapley Additive exPlanations (SHAP) were applied to the XGBoost model (20). SHAP summary plots were used to assess the global importance of individual features, while a waterfall-like visualization was generated to illustrate feature contributions for a representative high-risk individual.

## 3 Results

### 3.1 Baseline characteristics of the study population

**Table 1** summarizes the baseline characteristics of a total of 14,562 male participants, including 423 PCa patients and 14,139 non-PCa controls. The prevalence of hypertension did not differ significantly between the PCa and control groups. However, significant differences were identified between the two groups in age, race, educational level, BMI, smoking history, alcohol use, and the prevalence of diabetes. Compared to the control group, the PCa group was older (P trend< 0.0001), had a higher educational attainment (P = 0.0011), higher BMI (P = 0.0149), higher rates of smoking (P = 0.0048) and alcohol use (P = 0.0212), and a higher prevalence of diabetes (P = 0.0312). With regard to race/ethnicity, non-Hispanic White individuals appeared to have the highest risk of PCa (**Table 1**).

**Table 1:** Baseline characteristics participants with and without prostate cancer in NHANES 1999 – 2018.

Variable/n(%)	Cases (unweighted n = 423; weighted n = 1455361)	Controls (unweighted n = 14139; weighted n = 149697710)	Test of significance (χ2 value)	*P*a	Multivariable regression OR (95% CI)	*P*-multiv	*P*-trend
Age	70.86 ± 0.55	45.11 ± 0.20			1.12 (1.11-1.13)		<0.0001
Race			8.8271	<0.0001			
Mexican American	18 (4.3%)	2802 (19.8%)			1(Ref)	0.0145	
Non-Hispanic White	240 (56.7%)	6546 (46.3%)			2.70 (1.66-4.41)		
Non-Hispanic Black	124 (29.3%)	2767 (19.6%)			4.72 (2.76-8.09)		
Other Hispanic	23 (5.4%)	1036 (7.3%)			2.42 (1.20-4.87)		
Other Race	18 (4.3%)	988 (7.0%)			3.63 (1.51-8.73)		
Education			0.2371	0.6270		0.0011	
<HighSchool	132 (31.2%)	5122 (36.2%)			1(Ref)		
≥HighSchool	291 (68.8%)	9017 (63.8%)			1.61 (1.22-2.12)		
BMI			8.0060	0.0012		0.0149	0.0800
Normal	115 (27.2%)	3927 (27.8%)			1(Ref)		
Overweight	196 (46.3%)	5812 (41.1%)			1.87 (1.42-2.48)		
Obese	112 (26.5%)	4400 (31.1%)			1.69 (1.11-2.55)		
Any smoking history			17.6148	<0.0001		0.0048	
Yes	271 (64.1%)	8334 (58.9%)			1(Ref)		
No	152 (35.9%)	5805 (41.1%)			0.66 (0.49-0.87)		
Any alcohol use			0.0094	0.9230		0.0212	
Yes	363 (85.8%)	12718 (89.9%)			1(Ref)		
No	60 (14.2%)	1421 (10.1%)			0.62 (0.42-0.93)		
Diabetes			34.1116	<0.0001		0.0312	
Yes	92 (21.7%)	1703 (12.0%)			1(Ref)		
No	317 (74.9%)	12169 (86.1%)			0.67 (0.47-0.96)		
Borderline	14 (3.3%)	267 (1.9%)			1.00 (0.45-2.22)		
Hypertension			105.1163	<0.0001		0.179	
Yes	263 (62.2%)	4563 (32.3%)			1(Ref)		
No	160 (37.8%)	9576 (67.7%)			0.83 (0.64-1.08)		

BMI, body mass index; PCa, prostate cancer; OR, odds ratio; CI, confidence interval.

*P*a represents the P value for comparisons between PCa cases and controls using weighted t tests for continuous variables and weighted χ² tests for categorical variables.

*P*-multiv indicates the overall P value for each variable in the multivariable logistic regression model.

*P*-trend represents the P value for trend when variables were modeled as continuous or ordinal variables in the multivariable analysis.

With respect to peripheral blood parameters, PCa patients exhibited significantly lower lymphocyte counts, platelet counts, hemoglobin levels, and serum albumin compared to the control group (all P< 0.0001). Conversely, high-density lipoprotein cholesterol (HDL-C) (P< 0.0001) and C-reactive protein (CRP) (P = 0.0021) were significantly higher in the PCa group. Among the composite inflammatory indices, all indices showed statistically significant differences between the two groups, except platelet–albumin ratio (PAR) (P = 0.0573) and neutrophil–platelet ratio (NPR) (P = 0.0604) (all other P< 0.05; **Table 2**).

**Table 2:** Baseline differences in single inflammatory markers and composite inflammatory indices between participants with and without prostate cancer.

Variable/Median(IQR)	Cases (unweighted n = 423; weighted n = 1455361)	Controls (unweighted n = 14139; weighted n = 149697710)	Z_value	*P*
Neutrophil	3.90(1.80)	4.00(1.90)	-2.017	0.3350
Lymphocyte	1.60(0.90)	2.00(0.90)	-12.529	<0.0001
Monocyte	0.60(0.20)	0.60(0.20)	0.048	0.2640
Platelet	218.00(78.00)	237.00(75.00)	-5.731	<0.0001
HDLC	49.00(18.50)	45.00(17.00)	3.946	<0.0001
Hemoglobin	143.00(18.00)	152.00(15.00)	-13.944	<0.0001
Albumin	42.00(4.00)	43.00(5.00)	-10.191	<0.0001
CRP	1.90(3.645)	1.70(3.00)	2.027	0.0021
SII	518.143(360.687)	461.250(312.752)	4.03	<0.0001
NLR	2.308(1.478)	1.958(1.145)	7.063	<0.0001
PLR	135.882(71.509)	117.778(55.125)	6.505	<0.0001
LMR	2.833(1.621)	3.600(1.767)	-12.514	<0.0001
NPR	0.018(0.009)	0.017(0.009)	0.825	0.0604
SIRI	1.357(1.168)	1.094(0.841)	6.122	<0.0001
PAR	5.256(1.847)	5.429(1.787)	-2.954	0.0573
CAR	0.045(0.091)	0.039(0.070)	2.488	0.0004
PNI	50.500(6.000)	54.000(6.500)	-14.533	<0.0001
CLR	1.194(2.239)	0.833(1.499)	5.418	<0.0001
HALP	43.298(26.362)	56.150(28.486)	-12.372	<0.0001
AISI	294.912(262.398)	255.760(226.636)	3.375	<0.0001
IBI	4.748(9.029)	3.269(6.611)	4.600	<0.0001
MHR	0.012(0.006)	0.012(0.007)	-3.236	0.0290

HDLC, high-density lipoprotein cholesterol; CRP, C-reactive protein; SII, systemic immune-inflammation index; NLR, neutrophil-to-lymphocyte ratio; PLR, platelet-to-lymphocyte ratio; LMR, lymphocyte-to-monocyte ratio; NPR, neutrophil-to-platelet ratio; SIRI, systemic inflammation response index; PAR, platelet-to-albumin ratio; CAR, C-reactive protein-to-albumin ratio; PNI, prognostic nutritional index; CLR, C-reactive protein-to-lymphocyte ratio; HALP, hemoglobin–albumin–lymphocyte–platelet index; AISI, aggregate index of systemic inflammation; IBI, inflammatory burden index; MHR, monocyte-to-HDL cholesterol ratio.

Values are expressed as median (IQR). Group comparisons were conducted using the Mann–Whitney U test.

To further characterize the differences between study populations, we compared the baseline characteristics of PCa patients in the NHANES cohort and the external hospital-based validation cohort (**Supplementary Table S1**). Significant differences were observed across multiple variables, including age, BMI, lifestyle factors (smoking and alcohol use), and comorbidities (all P< 0.05). Notably, patients in the external cohort exhibited lower BMI and lower prevalence of smoking and alcohol use, reflecting distinct population characteristics. These findings indicate substantial heterogeneity between the two cohorts.

### 3.2 Associations between composite inflammatory indices and PCa risk

**Figure 1:**
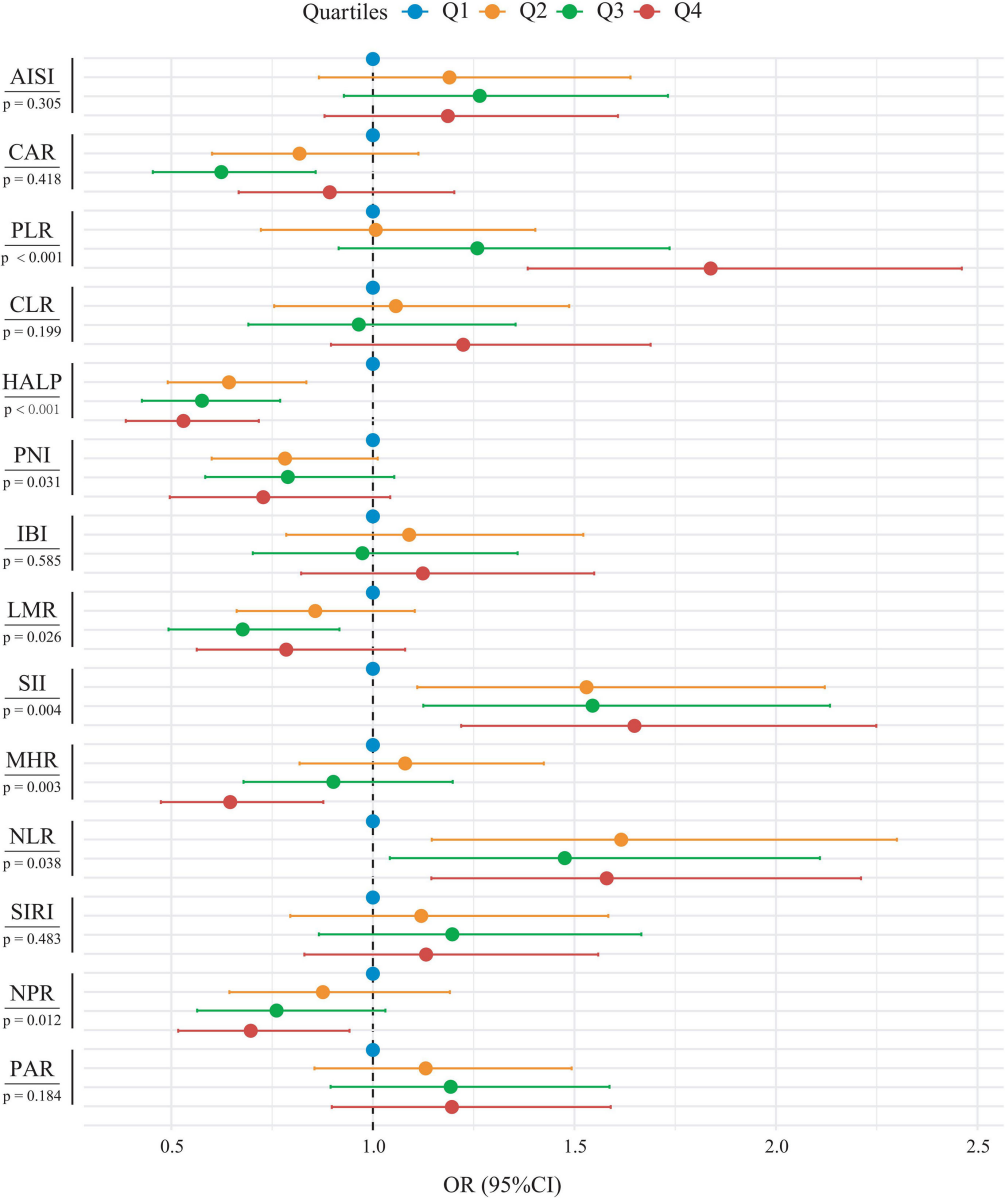
Forest plot showing the associations between composite inflammatory indices and prostate cancer risk. Inflammatory indices were categorized into quartiles (Q1–Q4), with the lowest quartile (Q1) as the reference. Odds ratios (ORs) and 95% confidence intervals (CIs) are presented. The vertical dashed line indicates the null value (OR = 1).

Given the skewed distribution of inflammatory indices, participants were categorized into quartiles (Q1–Q4), with the lowest quartile (Q1) serving as the reference group. Three progressively adjusted models were constructed according to covariate inclusion. In the crude model, most composite inflammatory indices were significantly associated with PCa risk. Higher SII, NLR, PLR, NPR, SIRI, CAR, CLR, AISI, and IBI were associated with an increased PCa risk. In contrast, elevated LMR, PNI, HALP, and MHR were associated with a decreased PCa risk. In the fully adjusted model 3, SII and PLR exhibited significant dose-response relationships with PCa risk, with odds ratios (ORs) increasing monotonically across quartiles (both P trend< 0.05). Conversely, NPR, HALP, and MHR showed significant inverse dose-response associations, with ORs decreasing progressively (P trend< 0.05 for all). Although the ORs did not change monotonically across quartiles, NLR remained positively associated with PCa risk, whereas LMR and PNI were inversely associated, with significant overall trends (**Table 3**). To visually illustrate the associations between composite inflammatory indices and PCa risk, **Figure 1** depicts the forest plot.

**Table 3:** Odds ratios (ORs) and 95% confidence intervals for prostate cancer across quartiles of composite inflammatory indices.

Item	Quartiles				*P* trend
	Q1	Q2	Q3	Q4	
AISI					
Range	<168.000	168.000–257.000	257.000–396.000	≥396.000	
Cases/Controls	77/3571	96/3541	111/3532	139/3495	
Crude OR (95%CI)	1	1.257 (0.929-1.707)	1.457 (1.088-1.962)	1.844 (1.395-2.456)	<0.001
Model 1 OR (95%CI)	1	1.094 (0.799-1.502)	1.154 (0.851-1.573)	1.100 (0.821-1.483)	0.538
Model 2 OR (95%CI)	1	1.186 (0.864-1.632)	1.275 (0.937-1.743)	1.204 (0.895-1.631)	0.244
Model 3 OR (95%CI)	1	1.190 (0.866-1.639)	1.265 (0.928-1.732)	1.186 (0.880-1.608)	0.305
CAR					
Range	<0.017	0.017–0.039	0.039–0.087	≥0.087	
Cases/Controls	90/3598	100/3495	91/3554	142/3492	
Crude OR (95%CI)	1	1.144 (0.857-1.528)	1.024 (0.762-1.376)	1.626 (1.246-2.132)	0.001
Model 1 OR (95%CI)	1	0.816 (0.603-1.105)	0.616 (0.453-0.840)	0.885 (0.669-1.176)	0.339
Model 2 OR (95%CI)	1	0.850 (0.627-1.155)	0.670 (0.490-0.915)	0.961 (0.724-1.281)	0.717
Model 3 OR (95%CI)	1	0.818 (0.601-1.113)	0.624 (0.454-0.858)	0.893 (0.667-1.202)	0.418
PLR					
Range	<93.333	93.333–118.182	118.182–148.818	≥148.818	
Cases/Controls	77/3575	78/3556	94/3541	174/3467	
Crude OR (95%CI)	1	1.018 (0.740-1.401)	1.233 (0.910-1.675)	2.330 (1.782-3.075)	<0.001
Model 1 OR (95%CI)	1	0.978 (0.704-1.359)	1.190 (0.868-1.635)	1.739 (1.315-2.319)	<0.001
Model 2 OR (95%CI)	1	0.983 (0.706-1.369)	1.214 (0.884-1.671)	1.756 (1.325-2.346)	<0.001
Model 3 OR (95%CI)	1	1.007 (0.722-1.403)	1.259 (0.915-1.736)	1.838 (1.384-2.461)	<0.001
CLR					
Range	<0.368	0.368–0.842	0.842–1.887	≥1.887	
Cases/Controls	64/3584	94/3545	103/3531	162/3479	
Crude OR (95%CI)	1	1.485 (1.080-2.055)	1.634 (1.195-2.249)	2.608 (1.956-3.518)	<0.001
Model 1 OR (95%CI)	1	1.028 (0.738-1.441)	0.925 (0.668-1.290)	1.182 (0.874-1.615)	0.269
Model 2 OR (95%CI)	1	1.072 (0.768-1.507)	1.007 (0.725-1.407)	1.273 (0.939-1.745)	0.113
Model 3 OR (95%CI)	1	1.057 (0.755-1.487)	0.965 (0.691-1.354)	1.224 (0.896-1.689)	0.199
HALP					
Range	<42.943	42.943–55.894	55.894–71.549	≥71.549	
Cases/Controls	207/3434	90/3550	67/3573	59/3582	
Crude OR (95%CI)	1	0.421 (0.326-0.539)	0.311 (0.234-0.409)	0.273 (0.202-0.364)	<0.001
Model 1 OR (95%CI)	1	0.632 (0.484-0.819)	0.570 (0.423-0.758)	0.523 (0.383-0.705)	<0.001
Model 2 OR (95%CI)	1	0.640 (0.489-0.831)	0.577 (0.428-0.770)	0.544 (0.398-0.735)	<0.001
Model 3 OR (95%CI)	1	0.643 (0.491-0.835)	0.576 (0.427-0.770)	0.530 (0.387-0.717)	<0.001
PNI					
Range	<50.500	50.500–53.500	53.500–57.000	≥57.000	
Cases/Controls	230/3613	90/3423	66/3800	37/3303	
Crude OR (95%CI)	1	0.413 (0.321-0.527)	0.273 (0.205-0.358)	0.176 (0.122-0.247)	<0.001
Model 1 OR (95%CI)	1	0.733 (0.563-0.946)	0.739 (0.549-0.984)	0.647 (0.442-0.923)	0.004
Model 2 OR (95%CI)	1	0.779 (0.598-1.007)	0.789 (0.584-1.053)	0.722 (0.492-1.033)	0.028
Model 3 OR (95%CI)	1	0.782 (0.600-1.012)	0.789 (0.584-1.053)	0.728 (0.496-1.043)	0.031
IBI					
Range	<1.322	1.322–3.304	3.304–8.037	≥8.037	
Cases/Controls	68/3575	99/3539	106/3534	150/3491	
Crude OR (95%CI)	1	1.471 (1.079-2.017)	1.577 (1.162-2.154)	2.259 (1.697-3.037)	<0.001
Model 1 OR (95%CI)	1	1.048 (0.759-1.456)	0.908 (0.660-1.256)	1.050 (0.778-1.430)	0.906
Model 2 OR (95%CI)	1	1.112 (0.803-1.549)	1.018 (0.738-1.414)	1.185 (0.874-1.619)	0.355
Model 3 OR (95%CI)	1	1.090 (0.785-1.522)	0.974 (0.702-1.359)	1.124 (0.822-1.549)	0.585
LMR					
Range	<2.800	2.800–3.600	3.600–4.571	≥4.571	
Cases/Controls	206/3476	103/3625	59/3475	55/3563	
Crude OR (95%CI)	1	0.479 (0.376-0.608)	0.286 (0.212-0.381)	0.260 (0.191-0.349)	<0.001
Model 1 OR (95%CI)	1	0.827 (0.641-1.063)	0.655 (0.478-0.885)	0.781 (0.563-1.068)	0.017
Model 2 OR (95%CI)	1	0.860 (0.665-1.107)	0.675 (0.491-0.914)	0.770 (0.553-1.058)	0.020
Model 3 OR (95%CI)	1	0.857 (0.662-1.104)	0.677 (0.493-0.917)	0.785 (0.563-1.080)	0.026
SII					
Range	<330.913	330.913–462.662	462.662–645.173	≥645.173	
Cases/Controls	70/3571	100/3540	109/3531	144/3497	
Crude OR (95%CI)	1	1.441 (1.060-1.969)	1.575 (1.165-2.142)	2.101 (1.579-2.820)	<0.001
Model 1 OR (95%CI)	1	1.424 (1.036-1.967)	1.374 (1.005-1.889)	1.480 (1.100-2.008)	0.026
Model 2 OR (95%CI)	1	1.515 (1.100-2.098)	1.515 (1.104-2.090)	1.639 (1.213-2.233)	0.004
Model 3 OR (95%CI)	1	1.530 (1.110-2.121)	1.545 (1.125-2.134)	1.649 (1.219-2.249)	0.004
MHR					
Range	<0.009	0.009–0.012	0.012–0.016	≥0.016	
Cases/Controls	118/3697	117/3402	106/3526	82/3514	
Crude OR (95%CI)	1	1.078 (0.831-1.398)	0.942 (0.721-1.229)	0.731 (0.548-0.971)	0.024
Model 1 OR (95%CI)	1	1.083 (0.824-1.422)	0.886 (0.670-1.170)	0.658 (0.488-0.884)	0.003
Model 2 OR (95%CI)	1	1.134 (0.861-1.493)	0.957 (0.722-1.267)	0.722 (0.534-0.973)	0.023
Model 3 OR (95%CI)	1	1.080 (0.818-1.424)	0.902 (0.679-1.198)	0.646 (0.474-0.877)	0.003
NLR					
Range	<1.476	1.476–2.000	2.000–2.633	≥2.633	
Cases/Controls	55/3589	101/3892	99/3185	168/3473	
Crude OR (95%CI)	1	1.693 (1.221-2.373)	2.028 (1.460-2.847)	3.157 (2.336-4.331)	<0.001
Model 1 OR (95%CI)	1	1.437 (1.024-2.036)	1.310 (0.931-1.861)	1.391 (1.014-1.933)	0.148
Model 2 OR (95%CI)	1	1.580 (1.121-2.246)	1.476 (1.044-2.108)	1.576 (1.143-2.204)	0.033
Model 3 OR (95%CI)	1	1.616 (1.146-2.300)	1.476 (1.042-2.109)	1.580 (1.145-2.211)	0.038
SIRI					
Range	<0.750	0.750–1.100	1.100–1.600	≥1.600	
Cases/Controls	66/3602	82/3549	117/3511	158/3477	
Crude OR (95%CI)	1	1.261 (0.910-1.754)	1.819 (1.345-2.480)	2.480 (1.863-3.338)	<0.001
Model 1 OR (95%CI)	1	1.008 (0.718-1.419)	1.058 (0.771-1.462)	1.049 (0.774-1.433)	0.712
Model 2 OR (95%CI)	1	1.109 (0.788-1.567)	1.218 (0.883-1.693)	1.168 (0.857-1.606)	0.333
Model 3 OR (95%CI)	1	1.120 (0.795-1.584)	1.197 (0.866-1.666)	1.132 (0.830-1.559)	0.483
NPR					
Range	<0.013	0.013–0.017	0.017–0.022	≥0.022	
Cases/Controls	92/3552	98/3544	112/3524	121/3519	
Crude OR (95%CI)	1	1.068 (0.800-1.426)	1.227 (0.928-1.626)	1.328 (1.010-1.751)	0.025
Model 1 OR (95%CI)	1	0.807 (0.596-1.093)	0.727 (0.541-0.977)	0.648 (0.485-0.868)	0.003
Model 2 OR (95%CI)	1	0.890 (0.656-1.209)	0.798 (0.592-1.077)	0.738 (0.549-0.993)	0.034
Model 3 OR (95%CI)	1	0.876 (0.644-1.191)	0.761 (0.564-1.031)	0.697 (0.517-0.942)	0.012
PAR					
Range	<4.600	4.600–5.425	5.425–6.395	≥6.395	
Cases/Controls	124/3518	105/3535	96/3552	98/3534	
Crude OR (95%CI)	1	0.843 (0.646-1.097)	0.767 (0.584-1.004)	0.787 (0.600-1.029)	0.057
Model 1 OR (95%CI)	1	1.095 (0.830-1.441)	1.153 (0.868-1.529)	1.143 (0.862-1.514)	0.301
Model 2 OR (95%CI)	1	1.130 (0.855-1.491)	1.187 (0.891-1.578)	1.193 (0.897-1.583)	0.192
Model 3 OR (95%CI)	1	1.131 (0.855-1.493)	1.193 (0.895-1.587)	1.196 (0.898-1.590)	0.184

BMI, body mass index; PCa, prostate cancer; OR, odds ratio; CI, confidence interval.

Q1 was used as the reference category. Model 1 was adjusted for age; Model 2 was adjusted for age, race, and education level; Model 3 was further adjusted for BMI, smoking history, alcohol use, hypertension, and diabetes.

*P* trend was calculated by modeling quartiles as an ordinal variable in the logistic regression model.

**Figure 2:**
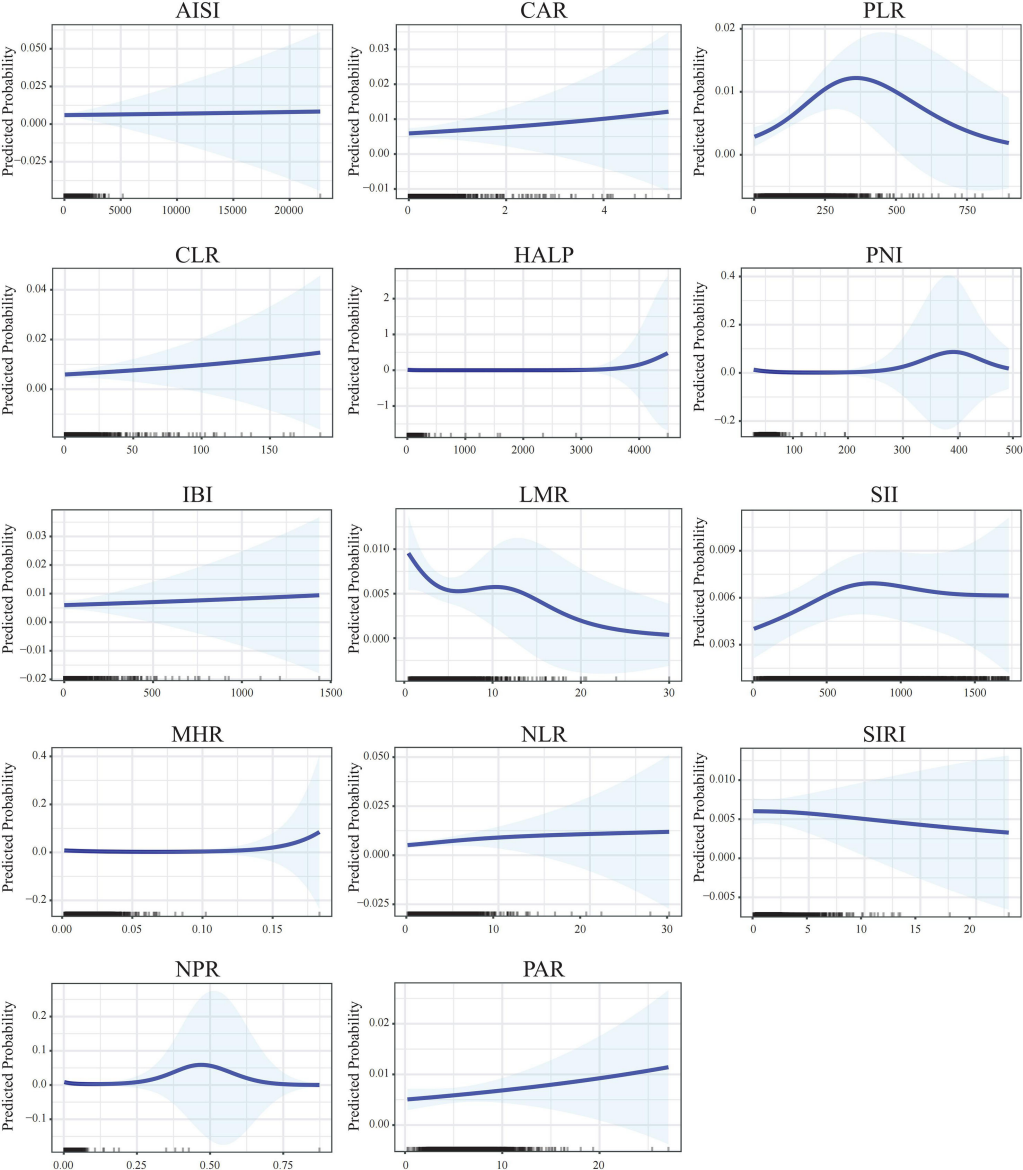
Generalized additive models illustrating the nonlinear associations between composite inflammatory indices and prostate cancer risk. The x-axis represents the levels of inflammatory indices, and the y-axis represents the estimated odds ratios. Solid lines indicate point estimates, and shaded areas represent 95% confidence intervals.

Subsequently, generalized estimating equation (GEE) models yielded associations that were largely consistent in direction with the primary analyses, although most did not reach statistical significance (**Supplementary Table S2**). Nonlinear analysis and *post hoc* power analysis suggested that several composite inflammatory indices exhibit nonlinear relationships with PCa risk, and comparisons between extreme quartiles indicated that most indices had adequate statistical power (**Supplementary Table S3**). To further characterize these associations, generalized additive models (GAMs) and smooth curve fitting were employed, revealing nonlinear relationships between multiple inflammatory indices and PCa risk (**Figure 2**).

### 3.3 Associations between composite inflammatory indices and mortality

Among the 423 PCa patients, follow-up data were available through December 2019, during which 185 deaths were recorded. Similar to the risk analyses, associations with all-cause mortality were evaluated using three progressively adjusted Cox proportional hazards models (**Table 4**). In the fully adjusted model, most inflammatory indices, including SII, NLR, PLR, LMR, PAR, PNI, HALP, and MHR, showed no significant dose-response relationships across quartiles (all P trend > 0.05). Multicollinearity among covariates included in the multivariable models was assessed using GVIF, and no significant multicollinearity was observed (**Supplementary Table S4**). However, NPR (P-trend = 0.0408), SIRI (P-trend = 0.0270), and AISI (P-trend = 0.0456) demonstrated significant associations, indicating that higher these indices were associated with an increased risk of death. Compared to the lowest quartile, the mortality risk was significantly increased in highest quartiles of CAR (hazard ratio [HR] = 2.21, 95% confidence interval [CI]: 1.44-3.41), CLR (HR = 1.70, 95% CI: 1.08-2.67), AISI (HR = 1.60, 95% CI: 1.03-2.48), and IBI (HR = 2.08, 95% CI: 1.33-3.27), though trend tests were not always significant. In addition, Kaplan–Meier survival analyses showed significant associations with all-cause mortality. As shown in **Supplementary Figure S1**, higher quartiles of NPR, SIRI, AISI, CAR, CLR, and IBI were consistently associated with poorer overall survival among PCa patients. The proportional hazards assumption was evaluated using the Schoenfeld residuals test, and visual inspection of the residual plots did not reveal major violations (**Supplementary Figure S2**).

**Table 4:** Hazard ratios (HRs) and 95% confidence intervals for all-cause mortality across quartiles of composite inflammatory indices among patients with prostate cancer.

Item	Quartiles				*P* overall	*P* Q4 *vs.*Q1	*P* trend
	Q1	Q2	Q3	Q4			
AISI							
Range	29.88–198.71	199.19–294.91	296.08–460.70	462.00–2509.53			
Number of deaths (%)	40 (37.7%)	50 (47.2%)	41 (39.0%)	54 (50.9%)			
Crude HR (95%CI)	1	1.25 (0.82–1.89)	1.05 (0.68–1.62)	1.83 (1.22–2.76)	0.0117	0.0038	0.0074
Model 1 HR (95%CI)	1	1.14 (0.75–1.73)	1.02 (0.66–1.57)	1.34 (0.89–2.04)	<0.0001	0.1653	0.0973
Model 2 HR (95%CI)	1	1.15 (0.75–1.74)	0.98 (0.63–1.52)	1.60 (1.03–2.48)	<0.0001	0.0347	0.0321
Model 3 HR (95%CI)	1	1.20 (0.79–1.82)	0.94 (0.60–1.47)	1.60 (1.03–2.48)	<0.0001	0.0361	0.0456
CAR							
Range	0.00–0.02	0.02–0.05	0.05–0.11	0.11–4.64			
Number of deaths (%)	39 (36.8%)	37 (34.9%)	56 (53.3%)	53 (50.0%)			
Crude HR (95%CI)	1	1.14 (0.72–1.78)	1.44 (0.96–2.17)	1.80 (1.19–2.72)	0.0269	0.0054	0.3730
Model 1 HR (95%CI)	1	1.23 (0.78–1.93)	1.52 (1.01–2.29)	2.23 (1.47–3.39)	<0.0001	0.0002	0.6070
Model 2 HR (95%CI)	1	1.22 (0.78–1.92)	1.50 (0.99–2.25)	2.24 (1.48–3.40)	<0.0001	0.0002	0.8424
Model 3 HR (95%CI)	1	1.22 (0.77–1.92)	1.40 (0.91–2.15)	2.21 (1.44–3.41)	<0.0001	0.0003	0.7964
PLR							
Range	1.65–103.85	105.50–135.88	136.11–176.00	176.36–401.25			
Number of deaths (%)	38 (35.8%)	50 (47.2%)	45 (42.9%)	52 (49.1%)			
Crude HR (95%CI)	1	0.99 (0.65–1.52)	0.94 (0.61–1.45)	1.11 (0.73–1.69)	0.8608	0.6124	0.4372
Model 1 HR (95%CI)	1	0.92 (0.60–1.41)	0.83 (0.54–1.28)	0.99 (0.65–1.50)	<0.0001	0.9451	0.8699
Model 2 HR (95%CI)	1	0.90 (0.59–1.37)	0.86 (0.56–1.33)	1.03 (0.67–1.56)	<0.0001	0.9058	0.7223
Model 3 HR (95%CI)	1	0.90 (0.58–1.38)	0.83 (0.54–1.29)	0.98 (0.64–1.50)	<0.0001	0.9201	0.8271
CLR							
Range	0.00–0.52	0.53–1.19	1.20–2.75	2.78–129.29			
Number of deaths (%)	36 (34.0%)	41 (38.7%)	58 (55.2%)	50 (47.2%)			
Crude HR (95%CI)	1	1.04 (0.66–1.62)	1.48 (0.97–2.24)	1.53 (0.99–2.34)	0.0816	0.0535	0.2747
Model 1 HR (95%CI)	1	0.97 (0.62–1.52)	1.56 (1.03–2.37)	1.72 (1.12–2.64)	<0.0001	0.0136	0.5201
Model 2 HR (95%CI)	1	0.97 (0.62–1.52)	1.54 (1.02–2.34)	1.73 (1.13–2.67)	<0.0001	0.0123	0.7533
Model 3 HR (95%CI)	1	0.98 (0.62–1.54)	1.49 (0.97–2.29)	1.70 (1.08–2.67)	<0.0001	0.0215	0.7566
HALP							
Range	8.39–32.24	32.42–43.30	43.66–58.66	58.73–4492.06			
Number of deaths (%)	54 (50.9%)	42 (39.6%)	50 (47.6%)	39 (36.8%)			
Crude HR (95%CI)	1	0.82 (0.55–1.23)	0.91 (0.62–1.34)	0.76 (0.50–1.15)	0.5738	0.1905	0.6420
Model 1 HR (95%CI)	1	0.83 (0.55–1.24)	0.92 (0.63–1.35)	0.89 (0.59–1.36)	<0.0001	0.6010	0.4325
Model 2 HR (95%CI)	1	0.80 (0.53–1.20)	0.89 (0.61–1.31)	0.86 (0.57–1.30)	<0.0001	0.4717	0.4695
Model 3 HR (95%CI)	1	0.76 (0.50–1.15)	0.95 (0.64–1.41)	0.92 (0.60–1.40)	<0.0001	0.6979	0.4344
PNI							
Range	31.50–47.50	48.00–50.50	51.00–53.50	54.00–403.50			
Number of deaths (%)	44 (40.7%)	62 (50.8%)	39 (43.3%)	40 (38.8%)			
Crude HR (95%CI)	1	1.15 (0.78–1.69)	0.77 (0.50–1.19)	0.85 (0.56–1.31)	0.2140	0.4656	0.1632
Model 1 HR (95%CI)	1	1.08 (0.74–1.60)	0.96 (0.62–1.49)	1.09 (0.70–1.68)	<0.0001	0.7080	0.3444
Model 2 HR (95%CI)	1	1.14 (0.77–1.70)	0.96 (0.61–1.49)	1.09 (0.70–1.69)	<0.0001	0.7006	0.3818
Model 3 HR (95%CI)	1	1.16 (0.78–1.73)	1.05 (0.66–1.64)	1.18 (0.76–1.85)	<0.0001	0.4608	0.3668
IBI							
Range	0.02–1.95	1.96–4.75	4.77–10.86	11.10–736.93			
Number of deaths (%)	34 (32.1%)	42 (39.6%)	55 (52.4%)	54 (50.9%)			
Crude HR (95%CI)	1	1.20 (0.76–1.89)	1.62 (1.06–2.49)	1.97 (1.28–3.03)	0.0081	0.0021	0.4097
Model 1 HR (95%CI)	1	1.11 (0.71–1.75)	1.66 (1.09–2.55)	2.01 (1.31–3.09)	<0.0001	0.0015	0.6517
Model 2 HR (95%CI)	1	1.13 (0.72–1.78)	1.64 (1.07–2.52)	2.08 (1.35–3.21)	<0.0001	0.0009	0.8816
Model 3 HR (95%CI)	1	1.11 (0.70–1.76)	1.57 (1.01–2.44)	2.08 (1.33–3.27)	<0.0001	0.0013	0.8841
LMR							
Range	0.86–2.11	2.12–2.83	2.86–3.71	3.75–13.00			
Number of deaths (%)	52 (48.6%)	47 (43.9%)	46 (44.7%)	40 (37.7%)			
Crude HR (95%CI)	1	0.77 (0.52–1.14)	0.72 (0.48–1.07)	0.68 (0.45–1.03)	0.2505	0.0717	0.0440
Model 1 HR (95%CI)	1	0.95 (0.64–1.42)	0.94 (0.63–1.41)	0.97 (0.64–1.48)	<0.0001	0.8903	0.5305
Model 2 HR (95%CI)	1	0.96 (0.64–1.43)	0.94 (0.63–1.40)	0.92 (0.61–1.41)	<0.0001	0.7161	0.4459
Model 3 HR (95%CI)	1	1.02 (0.68–1.55)	0.97 (0.64–1.47)	1.02 (0.67–1.57)	<0.0001	0.9170	0.5898
SII							
Range	8.89–379.08	379.75–518.14	518.32–739.20	741.00–2888.00			
Number of deaths (%)	38 (35.8%)	49 (46.2%)	48 (45.7%)	50 (47.2%)			
Crude HR (95%CI)	1	1.34 (0.87–2.04)	1.20 (0.79–1.84)	1.51 (0.99–2.30)	0.2747	0.0573	0.0448
Model 1 HR (95%CI)	1	1.30 (0.85–1.99)	1.14 (0.75–1.75)	1.27 (0.83–1.94)	<0.0001	0.2753	0.2277
Model 2 HR (95%CI)	1	1.33 (0.87–2.04)	1.13 (0.73–1.73)	1.38 (0.90–2.13)	<0.0001	0.1427	0.1523
Model 3 HR (95%CI)	1	1.41 (0.91–2.17)	1.15 (0.74–1.79)	1.35 (0.87–2.09)	<0.0001	0.1784	0.1779
MHR							
Range	0.00–0.01	0.01–0.01	0.01–0.02	0.02–0.18			
Number of deaths (%)	44 (40.0%)	47 (45.2%)	47 (42.3%)	47 (48.0%)			
Crude HR (95%CI)	1	1.12 (0.75–1.70)	1.01 (0.67–1.53)	1.41 (0.93–2.13)	0.3233	0.1056	0.8243
Model 1 HR (95%CI)	1	1.01 (0.67–1.52)	1.06 (0.70–1.59)	1.26 (0.83–1.91)	<0.0001	0.2717	0.4413
Model 2 HR (95%CI)	1	1.03 (0.68–1.55)	1.05 (0.69–1.60)	1.30 (0.85–1.99)	<0.0001	0.2184	0.5918
Model 3 HR (95%CI)	1	1.00 (0.65–1.52)	1.02 (0.67–1.57)	1.30 (0.84–2.00)	<0.0001	0.2359	0.5389
NLR							
Range	0.08–1.76	1.77–2.31	2.32–3.23	3.25–13.40			
Number of deaths (%)	37 (34.6%)	51 (48.6%)	43 (41.0%)	54 (50.9%)			
Crude HR (95%CI)	1	1.34 (0.88–2.05)	1.12 (0.72–1.73)	1.75 (1.15–2.66)	0.0399	0.0089	0.0207
Model 1 HR (95%CI)	1	1.02 (0.66–1.57)	0.89 (0.57–1.39)	1.26 (0.82–1.93)	<0.0001	0.2924	0.1434
Model 2 HR (95%CI)	1	0.97 (0.63–1.49)	0.86 (0.55–1.35)	1.33 (0.86–2.04)	<0.0001	0.1981	0.1279
Model 3 HR (95%CI)	1	0.97 (0.63–1.50)	0.84 (0.53–1.33)	1.27 (0.81–1.97)	<0.0001	0.2934	0.1916
SIRI							
Range	0.12–0.90	0.90–1.36	1.36–2.06	2.07–7.58			
Number of deaths (%)	40 (37.4%)	45 (42.5%)	44 (42.3%)	56 (52.8%)			
Crude HR (95%CI)	1	1.13 (0.74–1.73)	1.15 (0.75–1.76)	2.03 (1.35–3.06)	0.0016	0.0007	0.0011
Model 1 HR (95%CI)	1	0.95 (0.62–1.45)	0.92 (0.59–1.41)	1.41 (0.93–2.15)	<0.0001	0.1089	0.0428
Model 2 HR (95%CI)	1	0.94 (0.61–1.46)	0.92 (0.59–1.42)	1.60 (1.04–2.45)	<0.0001	0.0333	0.0104
Model 3 HR (95%CI)	1	0.92 (0.59–1.42)	0.88 (0.57–1.38)	1.48 (0.96–2.29)	<0.0001	0.0780	0.0270
NPR							
Range	0.01–0.01	0.01–0.02	0.02–0.02	0.02–0.35			
Number of deaths (%)	44 (41.5%)	42 (39.6%)	50 (47.6%)	49 (46.2%)			
Crude HR (95%CI)	1	0.97 (0.64–1.49)	1.31 (0.87–1.97)	1.53 (1.02–2.30)	0.0883	0.0412	0.0019
Model 1 HR (95%CI)	1	0.78 (0.51–1.19)	0.97 (0.65–1.46)	1.28 (0.85–1.93)	<0.0001	0.2399	0.0165
Model 2 HR (95%CI)	1	0.74 (0.48–1.13)	0.98 (0.65–1.48)	1.30 (0.86–1.96)	<0.0001	0.2143	0.0179
Model 3 HR (95%CI)	1	0.74 (0.48–1.13)	0.95 (0.62–1.44)	1.30 (0.85–1.99)	<0.0001	0.2265	0.0408
PAR							
Range	0.49–4.48	4.49–5.26	5.26–6.31	6.35–15.16			
Number of deaths (%)	45 (42.5%)	45 (42.5%)	42 (40.0%)	53 (50.0%)			
Crude HR (95%CI)	1	0.85 (0.56–1.29)	0.85 (0.56–1.30)	1.13 (0.76–1.68)	0.4351	0.5437	0.8387
Model 1 HR (95%CI)	1	0.84 (0.56–1.27)	0.83 (0.55–1.27)	1.23 (0.82–1.83)	<0.0001	0.3120	0.7003
Model 2 HR (95%CI)	1	0.87 (0.58–1.32)	0.85 (0.56–1.30)	1.27 (0.85–1.90)	<0.0001	0.2364	0.6566
Model 3 HR (95%CI)	1	0.84 (0.55–1.28)	0.89 (0.58–1.36)	1.23 (0.82–1.85)	<0.0001	0.3106	0.5322

Hazard ratios (HRs) and 95% confidence intervals (CIs) were estimated using Cox proportional hazards regression models. Inflammatory indices were categorized into quartiles (Q1–Q4) based on their weighted distribution in the study population, with the lowest quartile (Q1) serving as the reference group.

Crude model was unadjusted. Model 1 was adjusted for age; Model 2 was further adjusted for age, race, and education level; Model 3 was additionally adjusted for body mass index (BMI), smoking history, alcohol use, hypertension, and diabetes.

*P* overall, *P* Q4 vs. Q1, and *P* trend were calculated separately for each model. *P* overall represents the Wald test for the overall association of each inflammatory index with mortality across quartiles. *P* Q4 vs. Q1 indicates the statistical significance of the comparison between the highest and lowest quartiles. *P* trend was assessed by modeling the median value of each quartile as a continuous variable.

**Figure 3:**
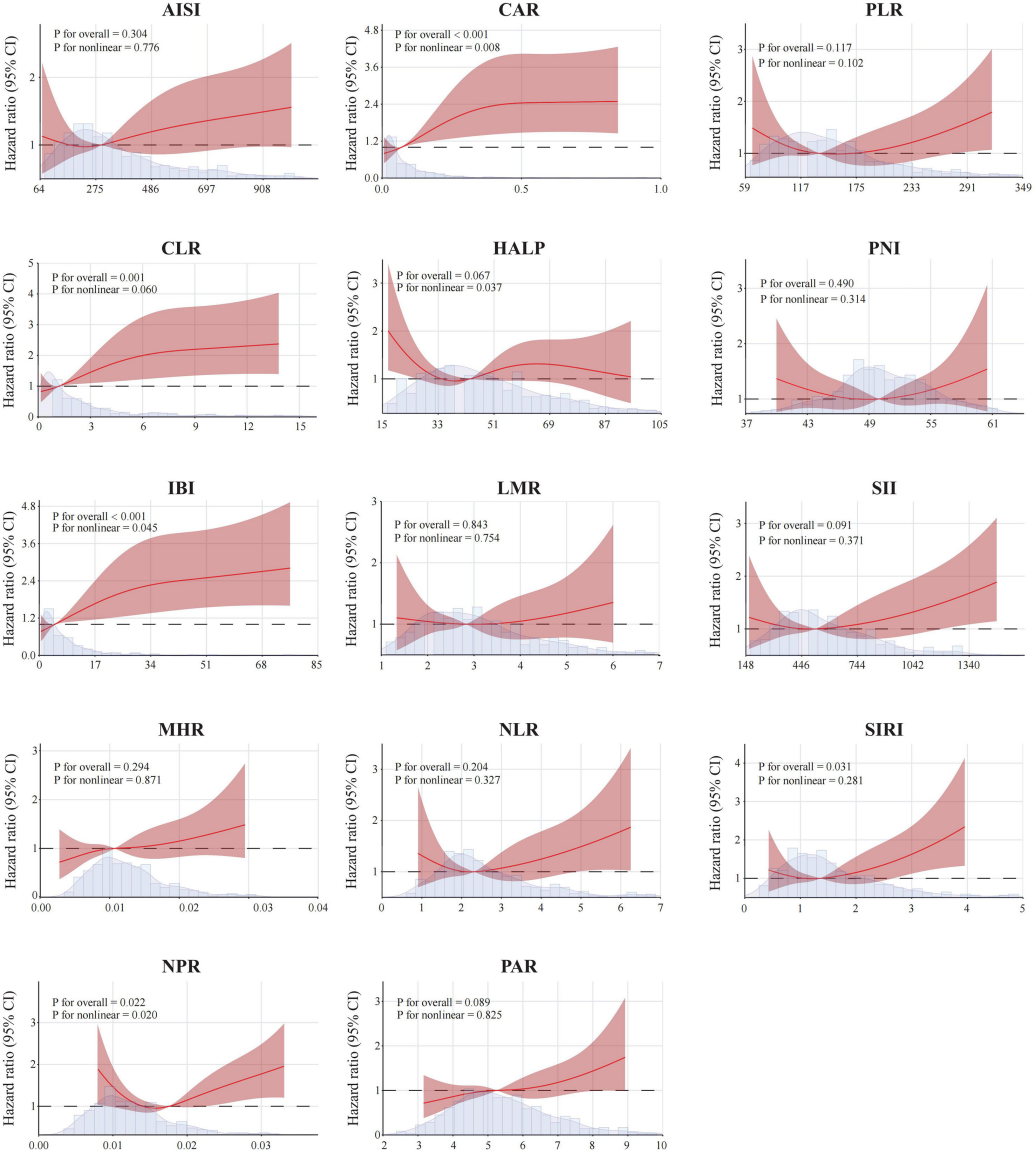
Restricted cubic spline (RCS) analyses showing the associations between composite inflammatory indices and all-cause mortality in patients with prostate cancer. Four knots were placed at the 5th, 35th, 65th, and 95th percentiles. Solid lines represent hazard ratios, and shaded areas indicate 95% confidence intervals. Models were fully adjusted for age, race, education level, body mass index, smoking history, alcohol use, hypertension, and diabetes.

**Figure 4:**
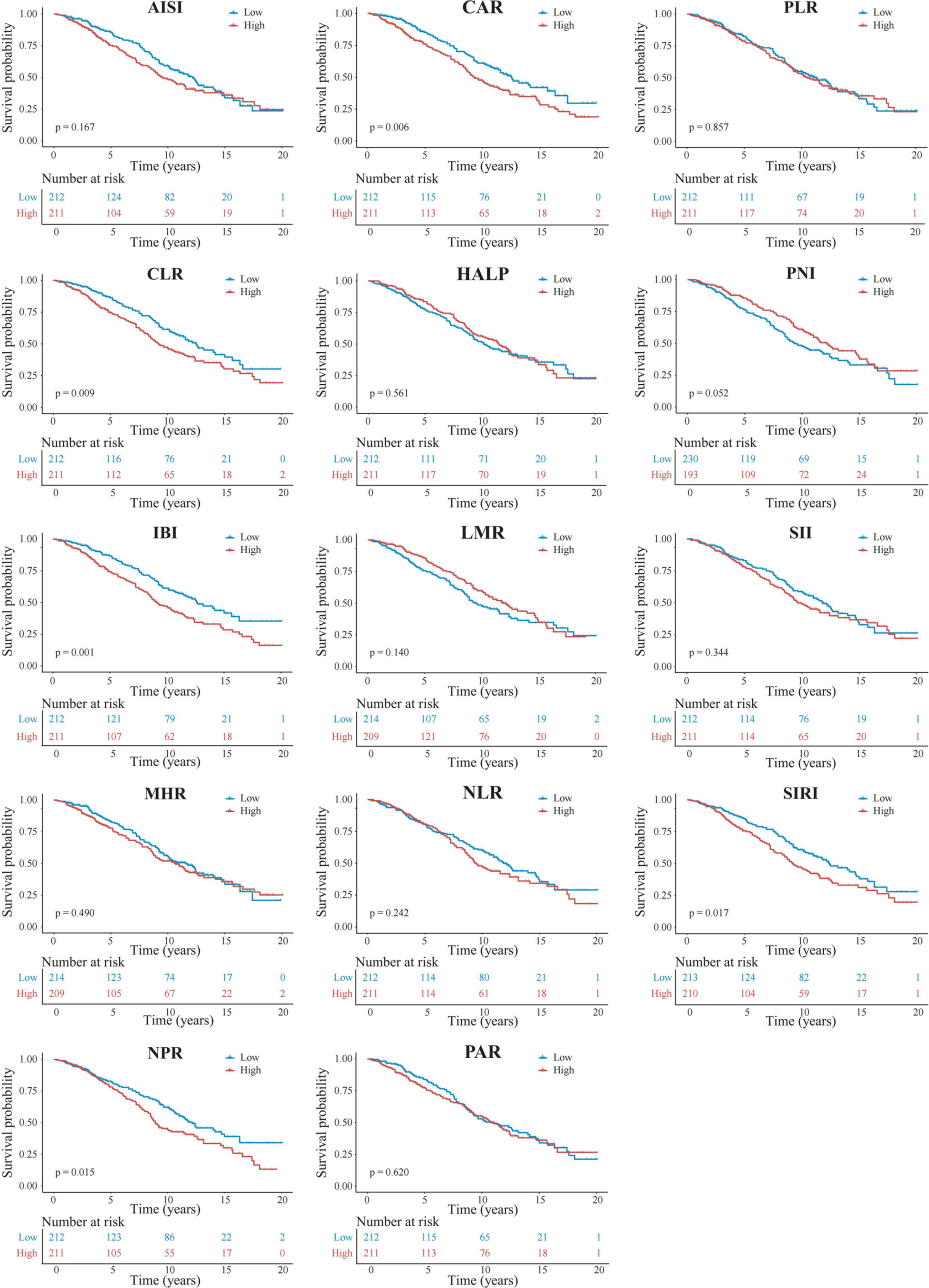
Kaplan-Meier survival curves comparing overall survival between high and low groups of composite inflammatory indices, stratified by median values. Differences between groups were assessed using the log-rank test.

Furthermore, in the fully adjusted model (Model 3), restricted cubic spline (RCS) analyses demonstrated nonlinear associations between most inflammatory indices and mortality risk (**Figure 3**). The RCS curves suggested that most inflammatory indices were nonlinearly associated with mortality risk, with distinct patterns observed across different value ranges. To further assess the prognostic relevance of these inflammatory indices, patients were divided into high and low groups based on the median values of each index, and Kaplan–Meier survival analyses were performed. The results demonstrated that higher CAR, CLR, IBI, SIRI, and NPR were consistently associated with poorer overall survival (all log-rank P< 0.05), suggesting their potential utility in risk stratification among PCa patients (**Figure 4**).

In summary, although not all inflammatory indices reached statistical significance in multivariable Cox proportional hazards models, the combined evidence from survival differences and nonlinear associations observed in RCS analyses indicates that these inflammatory indices may exert complex and threshold-dependent effects on PCa prognosis.

### 3.4 External validation of the predictive value of inflammatory indices

Based on the key findings derived from the NHANES data, we identified inflammatory indices that were consistently associated with PCa risk or prognosis, including SII, PLR, LMR, PNI, HALP, and NLR. These indices were subsequently validated in an independent hospital-based cohort from China. These indices were categorized into quartiles (Q1-Q4) according to each index, with the lowest quartile (Q1) serving as the reference (**Supplementary Table S5**). In the multivariable Cox models, after adjustment for potential confounding factors, SII, PLR, and NLR were significantly associated with all-cause mortality, with stable dose–response relationships observed for SII and PLR (P for trend< 0.05). Similarly, elevated NLR were significantly associated with adverse prognosis, with the highest quartile group having a higher mortality risk than the lowest quartile. In contrast, LMR, PNI, and HALP showed inverse associations with mortality risk, whereby higher these indices were generally associated with lower mortality risk; although some associations did not remain statistically significant after full adjustment for covariates.

**Figure 5:**
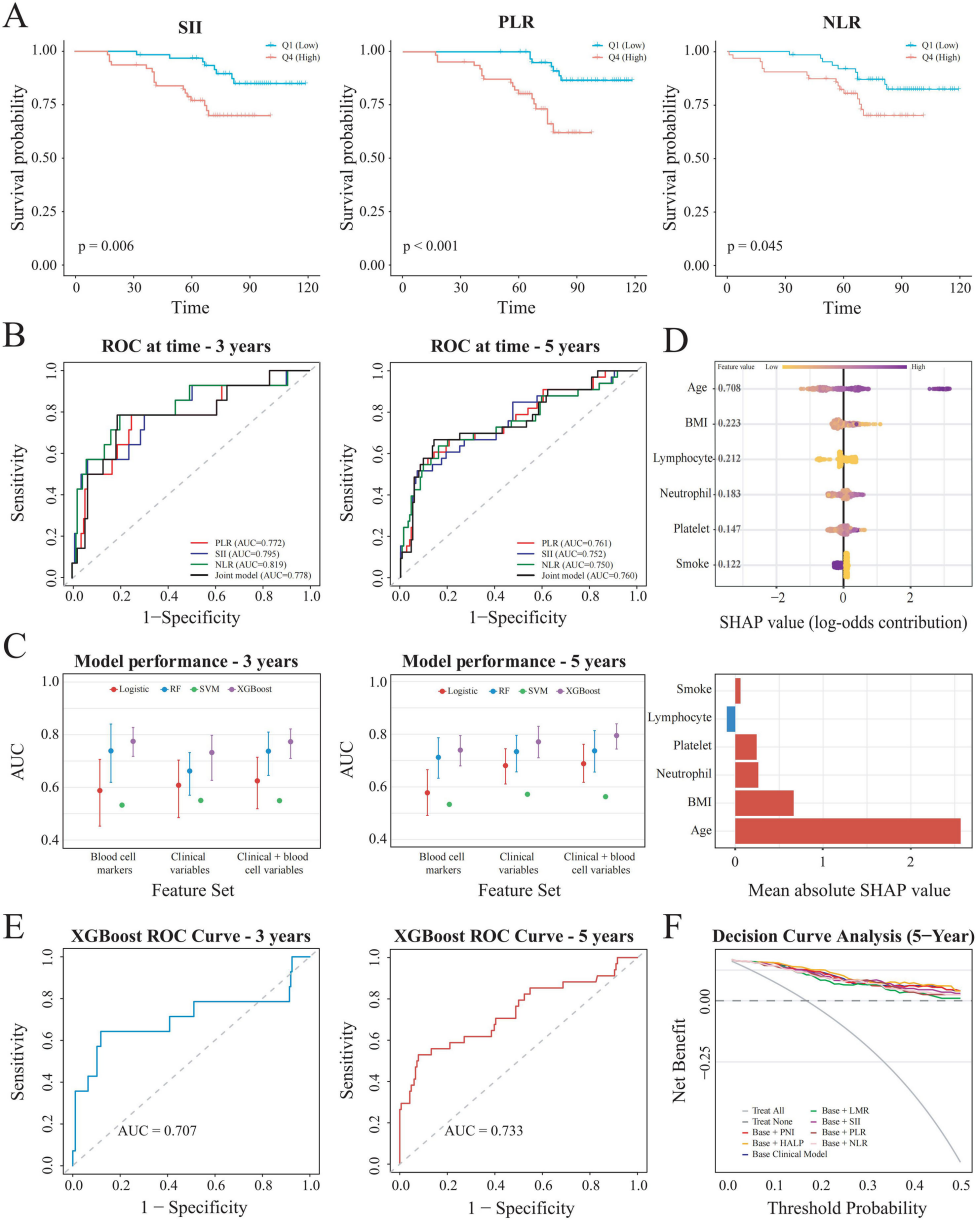
External validation and machine learning- based prediction of all-cause mortality in a hospital-based PCa cohort. **(A)** Kaplan-Meier survival curves comparing overall survival between the lowest (Q1) and highest (Q4) quartiles of SII, PLR, NLR. **(B)** Time-dependent ROC curves evaluating the predictive performance of SII, PLR, NLR, and their combined model for 3- and 5-year overall survival. **(C)** Comparison of time-dependent AUCs at 3 and 5 years across four machine learning models using different feature sets. **(D)** SHAP analysis of the XGBoost model showing feature contributions and relative importance. **(E)** Time-dependent ROC curves of the XGBoost model for predicting 3- and 5-year all-cause mortality in the external validation cohort. **(F)** Decision curve analysis (DCA) evaluating the incremental clinical net benefit of inflammatory indices when added to the clinical model in the external validation cohort.

Based on these findings, SII, PLR, and NLR, which exhibited the most robust and consistent associations with adverse prognosis, were selected as candidate prognostic indicators for further evaluation. Kaplan-Meier survival curves demonstrated significantly lower overall survival rates among patients in the highest quartile (Q4) compared with those in the lowest quartile (Q1) for all three indices (all log-rank P< 0.05) (**Figure 5A**). Time-dependent receiver operating characteristic (ROC) analyses revealed that SII, PLR, and NLR had good prognostic predictive ability for both 3-year and 5-year survival (**Figure 5B**). Further adjustment for established clinical factors (age, PSA, Gleason score, TNM stage, and treatment) demonstrated that SII, PLR, and NLR remained independent predictors of all-cause mortality, with modest improvements in model performance (**Table 5**).

**Table 5:** Incremental prognostic value of inflammatory indices for mortality in the external hospital-based cohort.

Variable HR (CI), *P*	Clinical model	+SII	+PLR	+NLR
Age	1.05 (1.01-1.10), 0.009	1.06 (1.02–1.11), 0.003	1.06 (1.02–1.11), 0.003	1.06 (1.02–1.11), 0.003
PSA	3.09 (1.26-7.61), 0.014	3.05 (1.21–7.66), 0.018	3.03 (1.21–7.63), 0.018	3.07 (1.22–7.70), 0.017
Gleason score (≥8 vs ≤7)	4.93 (1.41-17.23), 0.012	5.09 (1.44–18.00), 0.012	5.08 (1.45–17.80), 0.011	5.05 (1.43–17.85), 0.012
T stage (≥T3 vs T1–2)	26.03 (7.07-95.82), <0.001	26.81 (7.22–99.50), <0.001	27.43 (7.43–101.26), <0.001	27.29 (7.34–101.42), <0.001
N stage (N1 vs N0)	0.94 (0.41-2.16), 0.890	0.95 (0.42–2.17), 0.904	0.94 (0.41–2.13), 0.874	0.94 (0.41–2.16), 0.887
M stage (M1 vs M0)	1.27 (0.63-2.57), 0.503	1.39 (0.68–2.83), 0.368	1.37 (0.67–2.78), 0.390	1.37 (0.67–2.78), 0.387
Radiotherapy (Yes vs No)	1.08 (0.62-1.89), 0.775	1.15 (0.65–2.01), 0.634	1.14 (0.65–1.99), 0.651	1.14 (0.65–2.00), 0.640
Chemotherapy (Yes vs No)	0.76 (0.42-1.40), 0.387	0.76 (0.41–1.39), 0.371	0.77 (0.42–1.41), 0.395	0.75 (0.41–1.38), 0.352
ADT (Yes vs No)	0.69 (0.38-1.25), 0.220	0.60 (0.32–1.11), 0.106	0.61 (0.33–1.12), 0.109	0.61 (0.33–1.13), 0.117
Inflammatory index	-	1.00 (1.00–1.00), 0.021	1.00 (1.00–1.01), 0.012	1.10 (1.02–1.19), 0.015

Hazard ratios (HRs) and 95% confidence intervals (CIs) were derived from multivariable Cox proportional hazards regression models in the external validation cohort.

The clinical model included age, prostate-specific antigen (PSA), Gleason score, T stage, N stage, M stage, radiotherapy, chemotherapy, and androgen deprivation therapy (ADT).

To assess incremental prognostic value, each inflammatory index (SII, PLR, and NLR) was added separately to the clinical model.

All inflammatory indices were treated as continuous variables.

In summary, the validation cohort results further validated the potential prognostic value of SII, PLR, and NLR in PCa patients, supporting their utility as stable inflammation-related prognostic indicators.

### 3.5 Machine learning-based predictive performance

Building upon the composite inflammatory indices identified in the preceding analyses, we further examined whether their underlying hematological components could be used in machine learning to improve mortality risk prediction. Given that SII, PLR, and NLR share overlapping blood cell components, neutrophil, lymphocyte, and platelet were therefore selected as core features, together with clinical variables. Multiple machine learning models (including logistic regression, RF, SVM, and XGBoost) were constructed using different feature sets. Time-dependent ROC analyses demonstrated that models incorporating both clinical variables and peripheral blood parameters consistently outperformed those based on a single feature category at both 3-year and 5-year time points (**Figure 5C**).

Among all evaluated models, XGBoost achieved the highest and most stable AUC values. Given its superior predictive performance, XGBoost was selected for subsequent model interpretability analysis using shapley additive explanations (SHAP). As shown in **Figures 5D**, age, neutrophil, lymphocyte, platelet, BMI, and smoke history emerged as the most influential contributors to model-predicted mortality risk. Age and neutrophil were associated with higher predicted risk, whereas the effects of lymphocyte and platelet were heterogeneous and context dependent. To assess model generalizability, the trained XGBoost model was further applied to an independent external validation cohort. Time-dependent ROC analyses demonstrated good discrimination ability, with AUCs of 0.707 at 3 years and 0.733 at 5 years (**Figure 5E**). The concordance index (C-index) of the model was 0.640 in the external validation cohort, supporting the robustness of the machine learning-based prediction model.

Finally, decision curve analysis (DCA) was performed to assess the potential clinical utility of the model. As shown in **Figure 5F**, the model incorporating inflammatory indices demonstrated a modest increase in net clinical benefit compared with the clinical model across a range of threshold probabilities, suggesting a limited but measurable incremental clinical value in decision support.

## 4 Discussion

In this study, we systematically evaluated the associations between multiple composite inflammatory indices and PCa risk and mortality using the NHANES data, with an independent hospital-based cohort. Our findings demonstrate that several inflammatory indices are significantly associated with PCa risk or prognosis; however, the direction and pattern of these associations varied across different indices, highlighting the complex and nonlinear relationships. Specifically, elevated SII, PLR, and NLR, as well as decreased NPR, HALP, and MHR, were significantly associated with an increased risk of PCa. In contrast, higher NPR, SIRI, AISI, CAR, and CLR were associated with poorer prognosis among PCa patients. When combined with the results from the external cohort, we ultimately confirmed that elevated SII, PLR, and NLR consistently predicted poorer survival outcomes in PCa patients. Notably, substantial heterogeneity exists between the NHANES cohort and the external hospital-based cohort, particularly in terms of disease stage at diagnosis and baseline characteristics. Despite these differences, the consistent prognostic value of SII, PLR, and NLR across both cohorts supports their robustness and potential generalizability.

Previous studies have indicated that inflammatory indices are closely involved in the occurrence and progression of PCa. SII is associated with PCa mortality and serves as an independent prognostic factor in metastatic castration-resistant prostate cancer (mCRPC) patients treated with docetaxel (21, 22). Moreover, in patients undergoing radical prostatectomy, elevated SII has been associated with higher Gleason scores, serving as a promising biomarker for predicting Gleason score upgrading (23). PLR has been consistently linked to unfavorable outcomes in multiple malignancies, including gastric, lung, PCa, and colorectal cancers (24–26). When combined with other clinical indicators, PLR can improve PCa detection rate (27, 28). NLR has been identified as an important predictor of increased all-cause and cardiovascular mortality in several cancers, such as breast cancer, non-melanoma skin cancer, colorectal cancer, and melanoma (29). In patients with metastatic PCa receiving systemic therapy, elevated NLR was associated with an increased overall risk of death (30). Our findings support and complement previous studies, providing a theoretical foundation for the use of SII, PLR, and NLR as indicators of PCa risk and prognosis.

Notably, several inflammatory indices (including HALP, PNI, LMR, and MHR) were significantly associated with PCa risk but failed to demonstrate consistent prognostic value. This discrepancy suggests that inflammatory indices may play differentiated roles at multiple stages of PCa. Compared with indices emphasizing inflammatory response and immune imbalance, HALP and PNI place greater emphasis on nutritional status, immune reserves, and metabolic level. Consequently, alterations in these indices may be more relevant to PCa susceptibility, rather than to tumor behavior or treatment response (31, 32).

Our study has demonstrated the associations of SII, PLR, and NLR with PCa risk and prognosis. It is noteworthy that these inflammatory indices share common components—neutrophils, lymphocytes, and platelets—suggesting that the specific roles of these blood cells in PCa require further exploration. Neutrophils are key players of acute inflammatory responses, and it exert dual roles in cancer. Upon recruitment by vascular endothelial growth factor, neutrophils can release matrix metalloproteinase-9 to facilitate angiogenesis (33). Conversely, blocking the CXCR2 pathway can inhibit tumor growth and angiogenesis (34). In addition, tumor-associated neutrophils have been reported to promote the progression and therapeutic resistance of hepatocellular carcinoma by recruiting macrophages and regulatory T cells (35). On the other hand, in the presence of monoclonal antibodies, Neutrophils can produce pro-inflammatory cytokines and chemokines to help reverse immunosuppressive tumor microenvironments (36). In PCa, neutrophils can mediate tumor cell killing to reduce tumor bone metastasis. However, PCa cells can reverse these anti-tumor effects by inducing excessive reactive oxygen species production, ultimately promoting tumor progression (37).

Lymphocytes play critical roles in inhibiting tumor initiation, progression, and metastasis. CD8+T cells and natural killer cells exert potent anti-tumor effects through the release of perforin and granzymes, and the induction of apoptosis (38). Additionally, lymphocytes secrete multiple cytokines to modulate macrophage function, enhance antigen presentation, and reverse immunosuppressive microenvironments (39). In contrast, decreased lymphocytes can lead to a pro-tumorigenic microenvironment that promotes angiogenesis, extracellular matrix remodeling, and immunosuppression (40). Furthermore, analyzing peripheral lymphocyte subsets and other clinical variables can effectively predict risk stratification in PCa (41). Platelets not only play crucial roles in coagulation but are increasingly recognized as key components of the immune system. Thrombosis is a major complication in cancer patients, and tumor cells can communicate with platelets, activating platelets and promoting thrombus formation (42). Moreover, tumor cells can induce platelet aggregation, and activated platelets secrete stromal cell-derived factor 1α (SDF-1α) to mobilize cancer stem cells (43). In androgen receptor-negative PCa cells, platelets can enhance tumor invasiveness by upregulating the expression of matrix metalloproteinases (44). Furthermore, elevated ratios of platelets to neutrophils have been associated with poor prognosis and treatment outcome in metastatic hormone-sensitive PCa, as well as predictive of abiraterone treatment outcomes (45, 46).

To further explore the predictive utility of inflammation-related hematological features, we performed an exploratory machine learning using multiple algorithms. Although XGBoost demonstrated the best overall performance, its discriminative ability remained moderate, with AUCs of 0.751 and 0.831 for 3- and 5-year outcomes in the training set, and 0.707 and 0.733 in the validation set, respectively. Notably, the lower AUC observed for 3-year prediction compared with 5-year prediction suggests that short-term outcomes may be more susceptible to unmeasured factors, treatment heterogeneity, or random variation, particularly in population-based datasets. These findings indicate that, within current framework, machine learning models did not substantially outperform traditional inflammation-based risk assessment. The machine learning models should be regarded as complementary analytical tools rather than independent predictive method. Decision curve analysis further demonstrated that adding inflammatory indices to the clinical model provided only a modest increase in net clinical benefit across a range of threshold probabilities, suggesting limited but measurable clinical utility. Consistently, multivariable analyses adjusting for established clinical factors also showed that SII, PLR, and NLR remained independent predictors, but only yielded modest improvements in overall model performance.

In summary, neutrophils, lymphocytes, and platelets play crucial and complex roles in PCa. Compared to single blood cell counts, composite inflammatory indices offer a more comprehensive reflection of the dynamic interactions between the body and the tumor. With the advancement of technologies such as single-cell sequencing, more accurate classification of blood cells may further enhance the predictive power of inflammation-based biomarkers. From a clinical application perspective, our findings suggest that composite inflammatory indices may serve as cost-effective and readily available tools for risk stratification and prognostic assessment in PCa. The integration of inflammatory indices with other predictive models may contribute to improved surveillance in high-risk populations and promote individualized treatment. Although combining multiple inflammatory indices (SII, PLR, and NLR) did not confer a clear advantage over individual indices in prognostic prediction, this finding may be explained by overlapping hematological components and the limited sample size. This suggests that future studies should explore more effective combinations of data to enhance prognostic performance.

The strengths of this study include the utilization of a large-scale, nationally representative dataset, with comprehensive adjustment for potential confounding factors. By applying flexible approaches, we were able to identify nonlinear associations between composite inflammatory indices and PCa. Moreover, the incorporation of an independent validation cohort from a different country enabled us to assess the robustness and reproducibility of the findings derived from the NHANES, thereby substantially enhancing the generalizability and reliability of our conclusions. However, several limitations should be acknowledged. First, the prognostic association was evaluated using all-cause mortality rather than prostate cancer-specific mortality (PCSM). The lack of PCSM data, together with the presence of competing risks from non-cancer causes of death, may attenuate the specificity of the observed associations with prostate cancer outcomes. Second, the NHANES data are cross-sectional in nature, and inflammatory indices were measured at a single time point after cancer diagnosis. Therefore, it remains unclear whether elevated inflammatory levels preceded tumor development or were a consequence of the existing malignancy. As such, causal inference cannot be established, and these indices should be interpreted as prognostic or associative markers rather than etiological risk factors. Third, the assessment of PCa status was based on self-reported data, which may introduce recall bias. Finally, although inflammatory indices demonstrated independent prognostic value, their incremental improvement in predictive performance and clinical net benefit was modest, suggesting that their utility should be considered complementary rather than standalone. Therefore, large-scale prospective studies with longitudinal follow-up are warranted to validate and extend our findings.

## 5 Conclusion

In conclusion, this study demonstrates that multiple composite inflammatory indices are associated with PCa risk and prognosis, with SII, PLR, and NLR showing the most consistent and reproducible prognostic value across both NHANES and an independent validation cohort. These findings underscore the important role of systemic inflammation in PCa progression and support the potential utility of inflammation-based biomarkers derived from routine laboratory parameters. Given their accessibility and low cost, SII, PLR, and NLR may serve as practical tools for risk stratification and prognostic assessment in PCa.

## Data Availability

The original contributions presented in the study are included in the article/**Supplementary Material**. Further inquiries can be directed to the corresponding authors.
