# Supplementary figures and images for: Comparative evaluation of composite inflammatory indices for predicting prostate cancer risk and mortality: a NHANES-based study with external validation
Source: Front Oncol. 2026 May 11;16:1830463. doi: 10.3389/fonc.2026.1830463 (PMC13199051; doi:10.3389/fonc.2026.1830463)

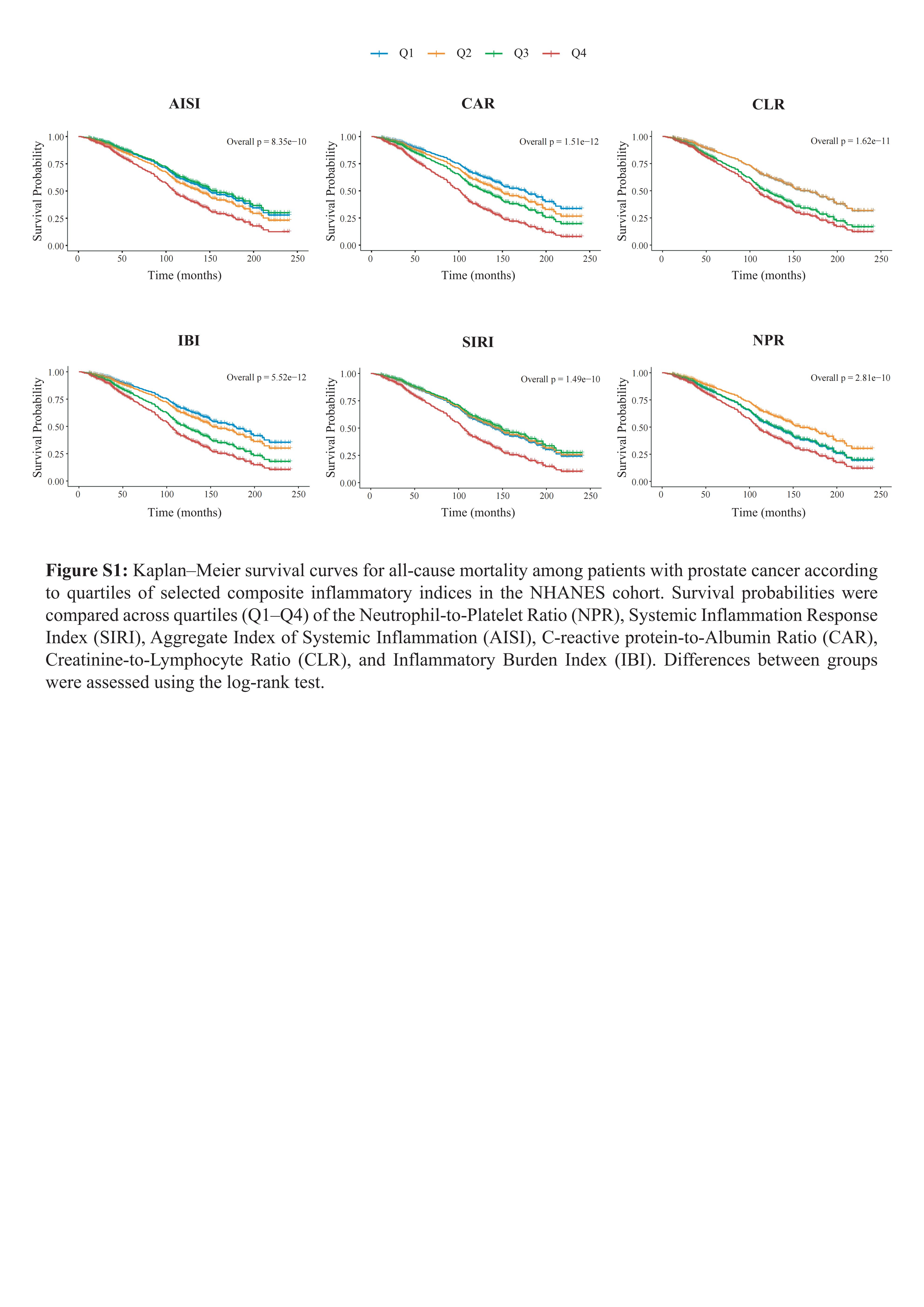

Supplement: Supplementary file 1 [file Image1.jpeg]

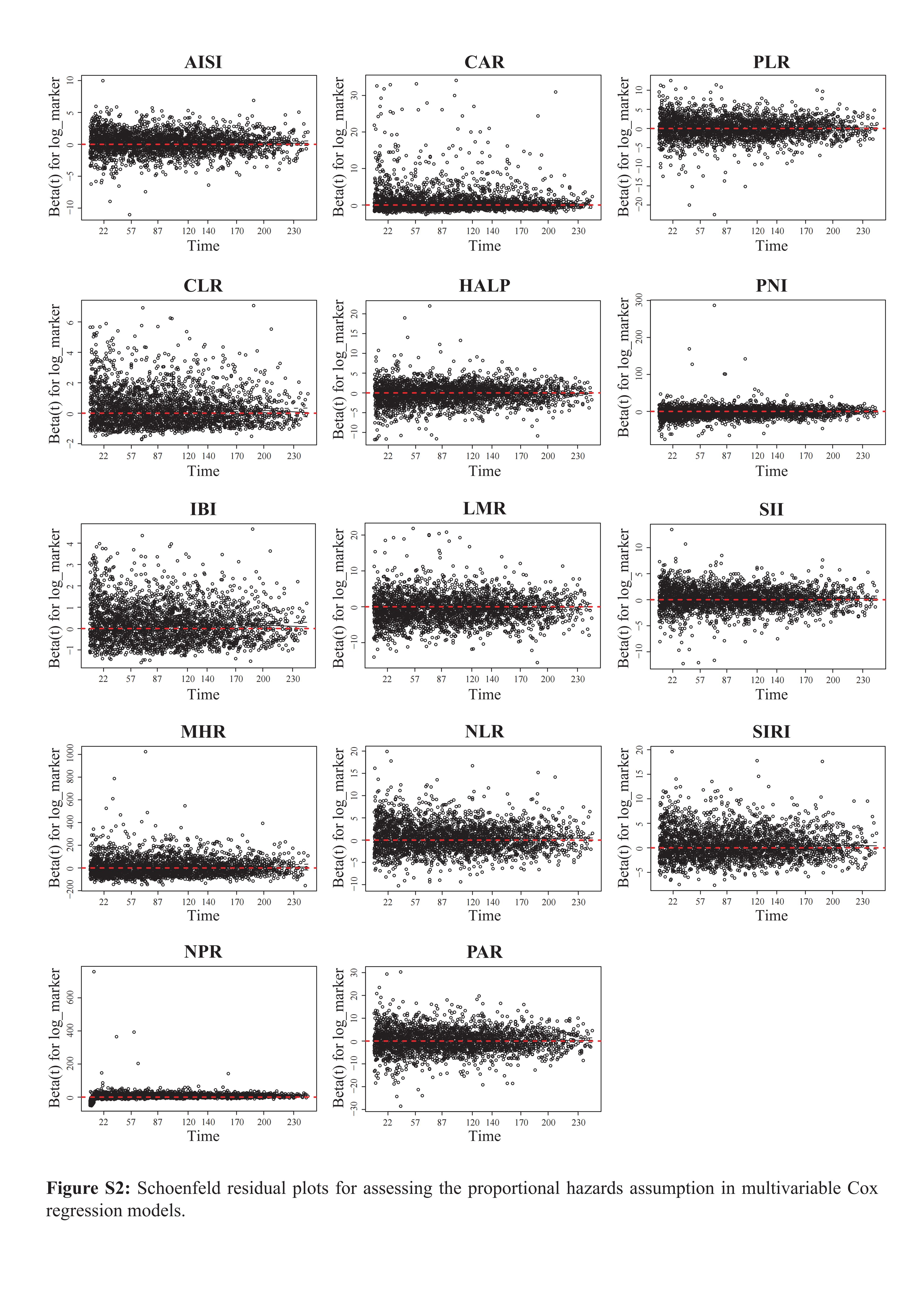

Supplement: Supplementary file 2 [file Image2.jpeg]

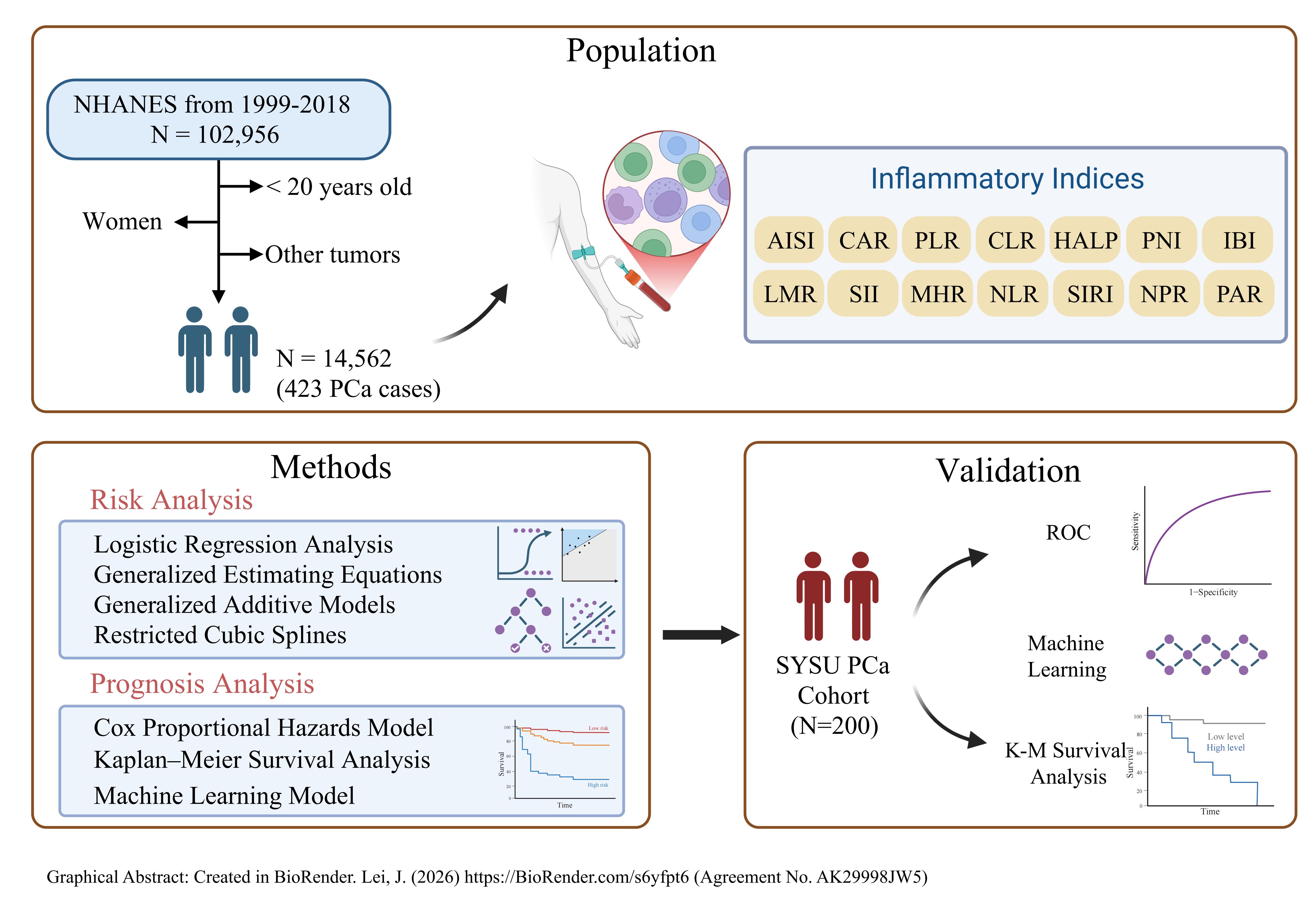

Supplement: Supplementary file 3 [file Image3.jpeg]
